# Outcomes of Myomectomy at the Time of Cesarean Section among Pregnant Women with Uterine Fibroids: A Retrospective Cohort Study

**DOI:** 10.1155/2019/7576934

**Published:** 2019-03-10

**Authors:** Rong Zhao, Xin Wang, Liying Zou, Weiyuan Zhang

**Affiliations:** Department of Obstetrics, Beijing Obstetrics and Gynecology Hospital, Capital Medical University, Beijing 100026, China

## Abstract

**Objective:**

A retrospective study was performed to evaluate the safety and feasibility of cesarean myomectomy among pregnant women with uterine fibroids (UFs).

**Methods:**

Upon data collection, the pregnant women with UF underwent cesarean section in the 39 hospital divided into two groups: cesarean myomectomy group, receiving cesarean section and myomectomy; cesarean group, receiving cesarean section only. Information about the type, location, and number of UFs was collected from the medical records or the prenatal ultrasound examinations.

**Results:**

In the cesarean myomectomy group, the proportion of subserous UFs was significantly higher than the cesarean group (65.6% versus 49.3%, P < 0.0001). The comparison of postpartum hemorrhage, neonatal weight, fetal distress, and neonatal asphyxia showed no statistical significance. Multivariate logistic regression analysis demonstrated that birth weight ≥4000 g (OR 3.1, 95% CI:1.6–6.0) and presence of diameter > 5 cm fibroids (OR 2.2, 95%CI:1.3–4.0) were high risk factors for PPH ≥1,000 ml.

**Conclusions:**

Myomectomy during cesarean section was a common procedure in mainland China. Myomectomy cesarean could be safe and feasible based on the estimation by experienced obstetricians. During the procedure, special attention should be paid to a large-sized leiomyoma ≥5cm and birth weight ≥4,000 g.

## 1. Introduction

Uterine fibroid (UF), a leiomyoma originating from the smooth muscle layer of uterus, is the most common benign tumor responsible for a major cause of morbidity in women of a reproductive age. To date, several factors have been reported as a consequence of UF, including uterine bleeding and pain, infertility, and spontaneous abortion [1–3]. Besides, various obstetric implications are also involved in the onset of UF such as breech presentation, preterm delivery, and placental abruption [4, 5].

Generally, most UF patients are asymptomatic and may not need therapy. However, a rapid progression may occur during pregnancy together with red degeneration. Nowadays, as more cases select cesarean section, more attention has been paid on whether to remove the fibroids at the time of cesarean section or not.

There are still some disputes on the myomectomy during the cesarean section as the procedure may raise the concerns about uncontrollable hemorrhage, which then brings about the necessity of hysterectomy and even elevation of postoperative morbidity, whereas some studies indicate that surgical management of UFs at cesarean section may be a safe option with careful case selection [6–8]. This study was designed to assess the effects of cesarean myomectomy on the obstetric outcomes.

## 2. Materials and Methods

### 2.1. Participants

This was a retrospective, multicentered, and cross-sectional study. Based on the multistage stratified random sampling method, the population involved in this survey included pregnant women with UFs received delivery in 39 hospitals chosen from 14 provinces in China mainland that could reflect the population distribution features. The study duration was from January 2011 to December 2011. This study protocols were approved by the Ethics Committees of medical institutions, respectively. The study design and performance were in line with the guidelines of the Helsinki Agreement. The National Research Ethics Service approved the anonymous use of these data for research purposes. Clinical information was collected, respectively, by reviewing patient's clinical medical recording files and the participants' personal information (e.g., name and address). Written informed consent was obtained from each participant. The data were analyzed anonymously.

### 2.2. Grouping and Data Collection

Upon data collection, the pregnant women with UF underwent cesarean sections in the 39 hospital divided into two groups: cesarean myomectomy group, receiving cesarean sections and myomectomy; cesarean group, receiving cesarean only.

The following maternal and fetal data were collected from each participant: maternal age, gravidity, parity, gestational age at delivery, weight, height, body mass index (BMI), history of smoking and/or drinking alcohol, presence of gestational diabetes metabolism (GDM), diabetes metabolism (DM), hypertensive disorder complicating pregnancy (HDCP), neonatal birth weight, Apgar scores (at 1 min, 5 min, and 10 min), fetal distress, blood loss, blood transfusion, type, location, size, and number of UF(s).

Information about the type, location, and number of UFs was collected from the medical records or the prenatal ultrasound examinations. The types of fibroids were divided into three categories including subserous, submucous, and intramural forms. The location was mainly in the corpus uterus, cervix, and lower uterus. The diameter of UF was divided into three categorized of <2cm, 2-5cm, >5cm, respectively. The number of UF was defined as single or multiple, respectively. In cases of multiple fibroids, the diameter of UF was defined as the diameter of the largest one. The size of the fibroid was based on the measurements made by the pathologists in the cesarean myomectomy group, while in the cesarean group, the size of the fibroid was defined based on the measurement of the prenatal ultrasound tests or estimation made by the surgeons recorded in the surgical report.

We further analyzed the high risk factors for postpartum hemorrhage ≥1,000 ml in the population. In order to rule out the influences of other interfering factors of postpartum hemorrhage, we excluded cases of placental abruption, placenta previa, coagulopathy, and cesarean section during labor stagnation. Univariate logistic regression analysis was performed to identify the possible risk factors for PPH ≥1,000 ml. Then the possible risk factors with P <0.2 were included in the multivariate logistic regression analysis to find out the high risk factors.

### 2.3. Statistical Analysis

SPSS 22.0 software was used for the statistical analysis. Enumeration data were presented as mean ± standard deviation. Measurement data were presented as percentages. Categorical variables were compared between groups using Student's t-test for parametric variables and Chi-square tests for nonparametric variables. P < 0.05 was considered to be statistically significant.

## 3. Results

### 3.1. Characteristics of Participants

In total, 2,565 women with UFs were included in this study. Among these participants, 2,344 (91.4%) underwent myomectomy during cesarean section and 221 (8.6%) underwent cesarean delivery only. The mean maternal age was 32.1 ± 5.0 yrs and 32.3 ± 5.2 yrs in cesarean myomectomy group and cesarean group, respectively. No statistical differences were noticed in mean maternal age, gestational age, parity, and BMI between the two groups (P>0.05, Table 1). The rate of drinker of cesarean alone group was higher than the cesarean myomectomy group (P= 0.002). While the rate of GDM, DM, and HDCP between the two groups showed no statistical difference (P>0.05), the rate of planned cesarean sections between the two groups also showed no statistical difference (P>0.05).

### 3.2. Comparison of UF Characteristics between Two Groups

In the cesarean myomectomy group, uterine fibroids less than 2 cm (43.6%) were the most common, followed by fibroids between 2 cm and 5 cm (43%) and fibroids more than 5 cm (13.4%). Meanwhile, there were 95 (43%) fibroids less than 2 cm, 101 (45.7%) fibroids between 2 cm and 5 cm and 25 (11.3%) fibroids more than 5 cm in the cesarean group. The proportion of UF with a diameter of > 5 cm in the cesarean myomectomy group showed no statistical difference compared to the cesarean group (13.4% versus 11.3%, P = 0.382). The proportion of subserous uterine fibroids was significantly higher in the cesarean myomectomy group than that of the cesarean group (65.6% versus 49.3%, P < 0.001). The proportion of intramural uterine fibroids was lower in the cesarean myomectomy group compared with the cesarean group (32.4% versus 46.6%, P < 0.0001). No statistical differences were noticed in the number of fibroids (P = 0.135, Table 2).

### 3.3. Comparison of Obstetric Outcomes between Two Groups

The mean blood loss in the cesarean myomectomy group was 835±42.3ml, while the mean blood loss in the cesarean alone group was 758±52.3ml. There is no statistical difference between the two group (P = 0.477) and no statistical difference was observed in the proportion of blood transfusion of the two group. There are also no statistical differences in other perioperative outcome variables between the two groups, including fetal distress, neonatal asphyxia, and neonatal birth weight (P>0.05, Table 3).

### 3.4. High Risk Factors for Postpartum Hemorrhage ≥1,000 ml

Univariate logistic regression analysis was first performed and the following possible risk factors were identified for postpartum hemorrhage ≥ 1,000 ml: age at delivery ≥ 35 yrs, maternal BMI ≥ 25, gestational week ≥ 37 weeks, primipara, and birth weight of newborn ≥ 4,000 g.

Multivariate logistic regression analysis demonstrated that birth weight≥4000 g (OR 3.1, 95% CI: 1.6–6.0) and presence of diameter≥5cm fibroids (OR 2.2, 95%CI: 1.3–4.0) were high risk factors for PPH ≥ 1,000 ml (Table 4).

After multivariate regression analysis, removal of fibroids during cesarean section did not increase the risk of postpartum hemorrhage (P=0.199). The type and location of fibroids during pregnancy had a certain effect on postpartum hemorrhage, but the difference was not statistically significant.

## 4. Discussion

The incidence of UFs in pregnant women shows rapid increase due to delay of child-bearing age in women. In clinical practice, obstetricians have to make a decision on whether to remove the fibroids during cesarean section or not. Conventionally, myomectomy is not recommended for those undergoing cesarean section as it may induce perioperative complications, especially the excessive hemorrhage that may involve an emergency hysterectomy [9–11]. In recent years, many studies indicated that myomectomy during cesarean delivery was safe and feasible [12–14]. We aim to investigate the safety and feasibility of cesarean myomectomy among pregnant women with uterine fibroids through analyzing the obstetric outcomes.

In a previous study [14], Kaymake et al. reported no significant increase of bleeding risk and frequency of blood transfusion between the patients underwent cesarean myomectomy or cesarean section alone. In addition, Topcu et al. [15] revealed the outcome variables (e.g., frequency of postoperative fever and mean duration of hospital stay) showed no significant difference between the cesarean myomectomy group and cesarean group. Moreover, myomectomy contributed to the change of hemoglobin levels despite the differences in the needed blood transfusions were not significant [16]. In this study, there were no differences in the mean blood loss and rate of blood transfusion between the patients underwent cesarean myomectomy and typical cesarean sections.

During myomectomy cesareans, specific techniques should be taken to lower the hemorrhage rate. For example, oxytocin should be administrated via intravenous or local injection. Furthermore, temporary ligation is performed to the uterine artery during myomectomy to decrease the intraoperative blood loss in those received myomectomy after suturing the uterine cesarean incision [13, 17, 18]. These results reflected the safety and feasibility of myomectomy during cesarean section for selected patients.

In this study, the proportion of subserous fibroids was significantly higher in the cesarean myomectomy group than that in the cesarean group, while the proportions of cervical and intramural fibroids were lower in the cesarean myomectomy group than that in the cesarean group. This indicated that myomectomy was performed more frequently in those with subserous fibroids, whereas cervical and intramural fibroids should be avoided during cesarean section. Besides, Kim et al. suggested that subserous fibroids should be removed during cesarean section [19]. Radmila S et al. recommended that pedunculated and subserosal myomas can be removed and intramural and multiple fibroids should be avoided [20]. Moreover, studies suggested that intramural fibroids myomectomy should be avoided in the presence of some complications [19, 21].

We further analyzed the high risk factors for postpartum hemorrhage ≥1,000 ml in pregnant women with uterine fibroids during cesarean section. Our data showed that the presence of a large-sized leiomyoma ≥5cm and birth weight ≥4,000 g were the important risk factors, while myomectomy during cesarean and the location and type of fibroids have little effects. In line with our study, Dedes I et al. reported that a larger size of fibroid (≥5 cm) and a maternal age more than 40 years are risk factors of increased blood loss in women with uterine fibroids during cesarean [22]. Sei K et al. investigated 759 pregnant women and reported the presence of a uterine fibroid of 175 cm^3^, birth weight of 2,500 g, and primipara were predictors for massive intraoperative hemorrhage during cesarean delivery [23].

Compared with the normal uterus myometrium, the blood flow rate in uterine fibroids and adjacent normal myometrium was decreased, which may decrease the distribution of oxytocin. The low distribution of oxytocin may result in uterine atony during cesarean [24]. Meanwhile the irregular lines in the entire myometrium may also inhibit muscular contraction of the uterus and cause uterine atony. The mechanism of the increased risk of hemorrhage with a macrosomia may be based on over-distended uterus and induced uterine atony [25, 26].

Although our study involving a reliable multicenter database included complete outpatient medical records and ward records, ultrasound findings, and operation reports, the present study still had several limitations. Ideally, a research with robust data should be based on a prospective randomized trial; however, such trials might have ethical problems. Thus, the present analysis was based on retrospective controlled study. In our study, we commented on short-term morbidity, while the long-term effects may have been missed. For example, the possible adverse effects on future pregnancies were not available.

## 5. Conclusions

In conclusion, our study indicated that myomectomy during cesarean section was a common procedure in mainland China. Myomectomy cesarean could be safe and feasible based on the estimation by experienced obstetricians. And during the procedure, presence of a large-sized leiomyoma ≥5cm and birth weight ≥4,000 g should be pay attention.

## Figures and Tables

**Table 1:** Sociodemographic and clinical characteristics of pregnant women with uterine fibroids taken cesarean.

Characteristics	Cesarean myomectomy group (n= 2344)	Cesarean alone group (n= 221)	P value
Mean maternal age (yr)	32.1 ± 5.0	32.3 ± 5.2	0.532
Mean Gestational age at delivery (wk)	38.4 ± 2.0	38.4 ± 2.1	0.752
Gravidity	1.9 ± 1.2	2.0 ± 1.2	0.283
Parity	1.2 + 0.4	1.2 ± 0.4	0.567
Body mass index (kg/m^2^)	22.5 ± 3.1	22.6 ± 2.7	0.749
Weight (kg)	58.8 ± 9.0	59.1 ± 7.8	0.701
Height (m)	1.6 ± 4.7	1.6 ± 4.9	0.172
Current smoker	0.3%	0.9%	0.145
Drinker	1.3%	4.1%	0.002
GDM^a^	12.8%	16.3%	0.146
DM^b^	0.3%	0	0.425
HDCP^c^	9.1%	8.1%	0.626
Planned cesarean	70.6	69.2	0.678

*Abbreviations*. ^a^GDM, gestational diabetes mellitus; ^b^DM, diabetes mellitus; ^c^HDCP, hypertensive disorder complicating pregnancy

**Table 2:** Characteristics of uterine fibroids in pregnant women taken cesarean.

Characteristics	Cesarean myomectomy group (n= 2344)	Cesarean alone group (n= 221)	P value
Size			
0-2 cm	1021(43.6%)	95(43%)	0.870
2-5 cm	1009(43%)	101(45.7%)	0.446
>5 cm	314(13.4%)	25(11.3%)	0.382
Location			
Corpus	2210(94.3)	204(92.3)	0.233
Lower segment	110(4.7)	9(4.1)	0.675
Cervix	24(1.0)	8(3.6)	0.001
Type			
Subserous	1537(65.6%)	109(49.3%)	0.0001
Submucous	48(2.0%)	9(4.1%)	0.051
Intramural	759(32.4%)	103(46.6%)	0.0001
Numbers			0.135
Single	1682(71.8%)	169(76.5%)	
Multiple	662(28.2%)	52(23.5%)	

**Table 3:** Obstetric outcomes of uterine fibroids in pregnant women taken cesarean section.

Outcomes	Cesarean myomectomy group (n= 2344)	Cesarean alone group (n= 221)	P value
Mean blood loss	835 ± 42.3	758 ± 52.3	0.477
Blood transfusion	20(0.9%)	4(2.1)	0.127
Fetal distress	150(6.4%)	16(7.2%)	0.627
Apgar<7 at 1 min	46(12)	4(1.8)	0.859
Apgar<7 at 5 min	13(0.6)	3(1.4)	0.156
Apgar<7 at 10 min	9(0.6)	1(0.5)	0.954
Neonatal birth weight (g)	3238±593.9	3241±578.1	0.133

**Table 4:** High risk factors for PPH ≥1000 ml by multivariate analysis.

Significant Predictors	OR	95% CI	P value
BMI ≥25	1.1	0.6-2.0	0.644
Primipara	0.6	0.3-1.2	0.15
Maternal age ≥35 years	1.4	0.8-2.3	0.224
Myomectomy during *cesarean*	1.6	0.8-3.1	0.199
Birth Weight ≥ 4000g	3.1	1.6-6.0	0.001
Diameter of uterine fibroid			0.019
Diameter ≥5cm vs <2cm	2.2	1.3-4.0	0.006
Diameter 2-5cm vs <2cm	2.2	1.0-4.7	0.039
Uterine fibroid types			0.772
Intramural vs Subserous	0.5	0.1-3.6	0.474
Submucous vs Subserous	1.0	0.6-1.6	0.896
Fibroids numbers (multi vs single)	1.0	0.6-1.7	0.994
Location of fibroids			0.276
Cervix vs Corpus	3.7	0.7-19.3	0.12
Lower segment vs Corpus	1.3	0.5-3.3	0.64

## Data Availability

The data used to support the findings of this study are available from the corresponding author upon request.

## Abbreviations

UFs: Uterine fibroids
BMI: Body mass index
GDM: Gestational diabetes metabolism
DM: Diabetes metabolism
HDCP: Hypertensive disorder complicating pregnancy.
